# Supplementary material for: Bifunctional Hot Water Vapor Template-Mediated Synthesis of Nanostructured Polymeric Carbon Nitride for Efficient Hydrogen Evolution
Source: Molecules. 2023 Jun 20;28(12):4862. doi: 10.3390/molecules28124862 (PMC10302706; doi:10.3390/molecules28124862)
Supplement: Supplementary file 1 [file molecules-28-04862-s001.zip › molecules-2450970-supplementary.pdf]

molecules

MDPI

Supplementary Materials

Bifunctional Hot Water Vapor Template-Mediated Synthesis of Nanostructured Polymeric Carbon Nitride for Efficient Hydrogen Evolution

Baihua Long [1], Hongmei He [1], Yang Yu [1], Wenwen Cai [1], Quan Gu [2], Jing Yang [3,*] and Sugang Meng [4,*]

[1] College of Material and Chemical Engineering, Pingxiang University, Pingxiang 337055, China
[2] Key Laboratory of Applied Surface and Colloid Chemistry, Ministry of Education, School of Chemistry and Chemical Engineering, Shaanxi Normal University, Xi'an 710062, China
[3] College of Health Science and Environmental Engineering, Shenzhen Technology University, Shenzhen 518118, China
[4] Key Laboratory of Green and Precise Synthetic Chemistry and Applications, Ministry of Education, Huaibei Normal University, Huaibei 235000, China
* Correspondence: yangjingsztu@163.com (J.Y.); mengsugang@126.com (S.M.); Tel.: +86-18156139968 (S.M.)

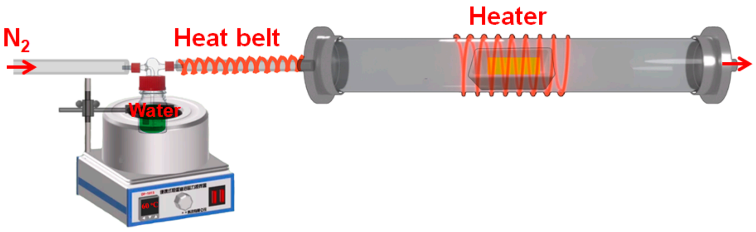

Scheme S1. Proposed one-step synthesis of nanostructured PCN by introducing hot water vapor into the polymerization process of C/N precursors.

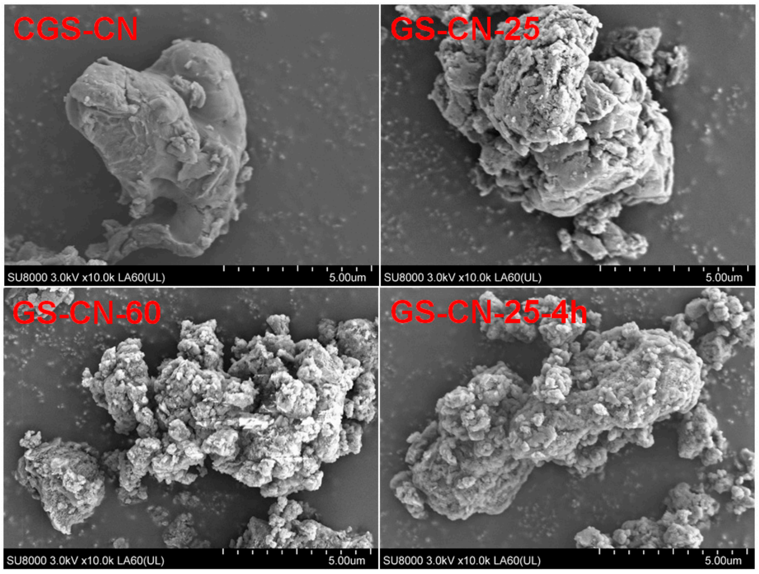

Figure S1. SEM images of bulk CGS-CN and GS-CN-x photocatalysts.

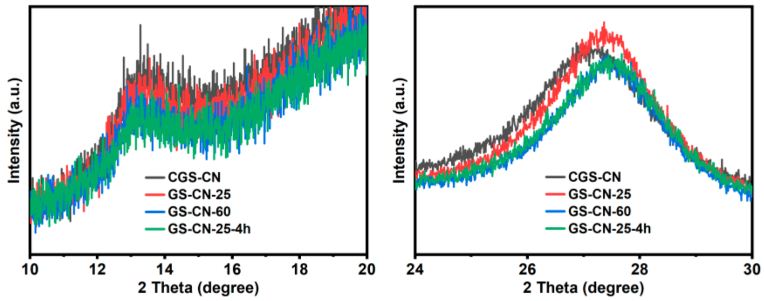

Figure S2. The high-magnification section of the XRD patterns for bulk CGS-CN and GS-CN-x photocatalysts.

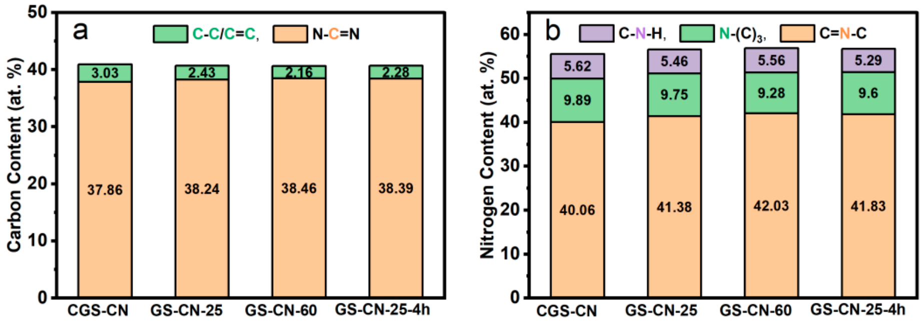

Figure S3. Summary of C atomic contents in the various photocatalysts determined by C1s spectra (a) and summary of N atomic contents in the various photocatalysts determined by N1s spectra (b).

Note: the values in the graph are obtained by calculating the percentage of the selected chemical bond's peak area to the corresponding element's total peak area based on XPS analysis results.

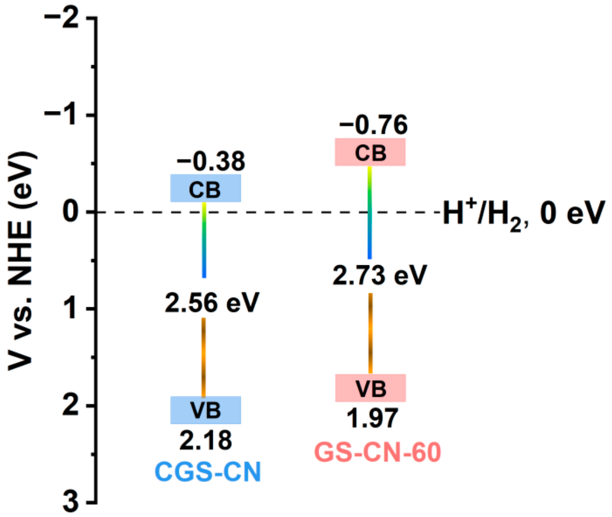

Figure S4. The energy band diagrams of bulk CGS-CN and GS-CN-60.

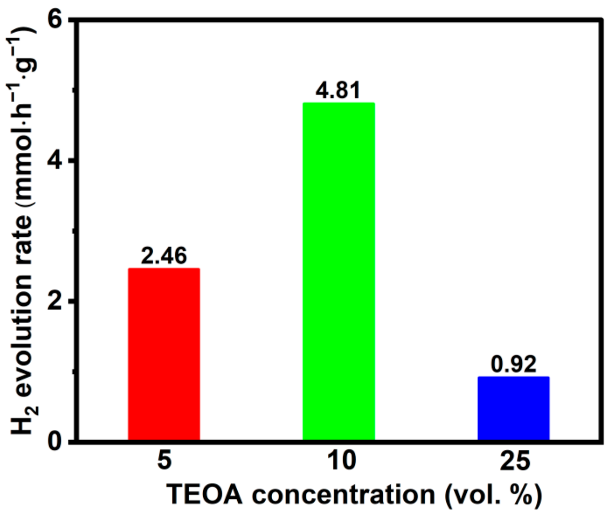

Figure S5. H_2 evolution rate from different concentration of TEOA as a sacrificial electron donor by GS-CN-60 photocatalyst.

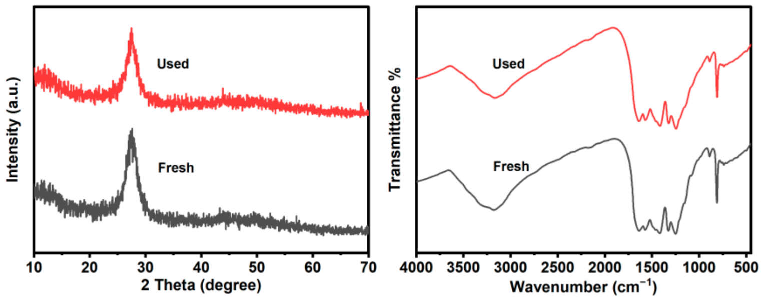

Figure S6. XRD and FT-IR patterns of the GS-CN-60 for photocatalytic H_2 evolution before and after five cycling runs.

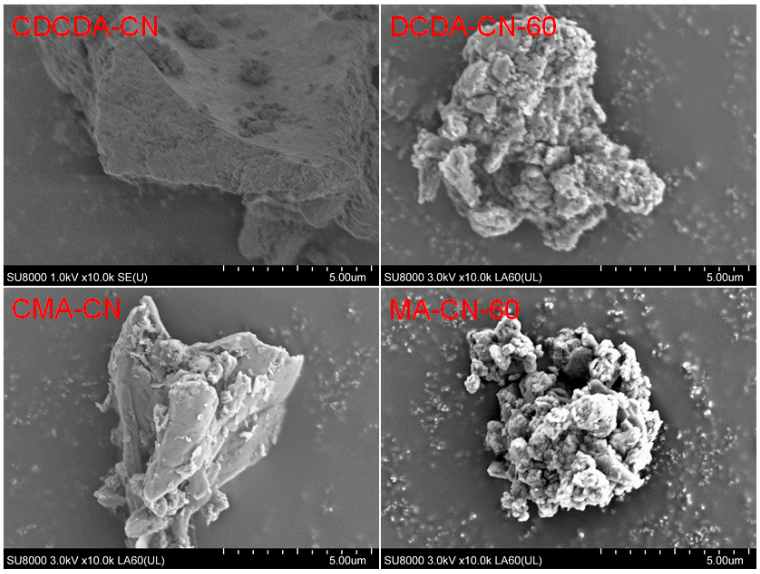

Figure S7. SEM images of CDCDA-CN, DCDA-CN-60, CMA-CN, and MA-CN-60.

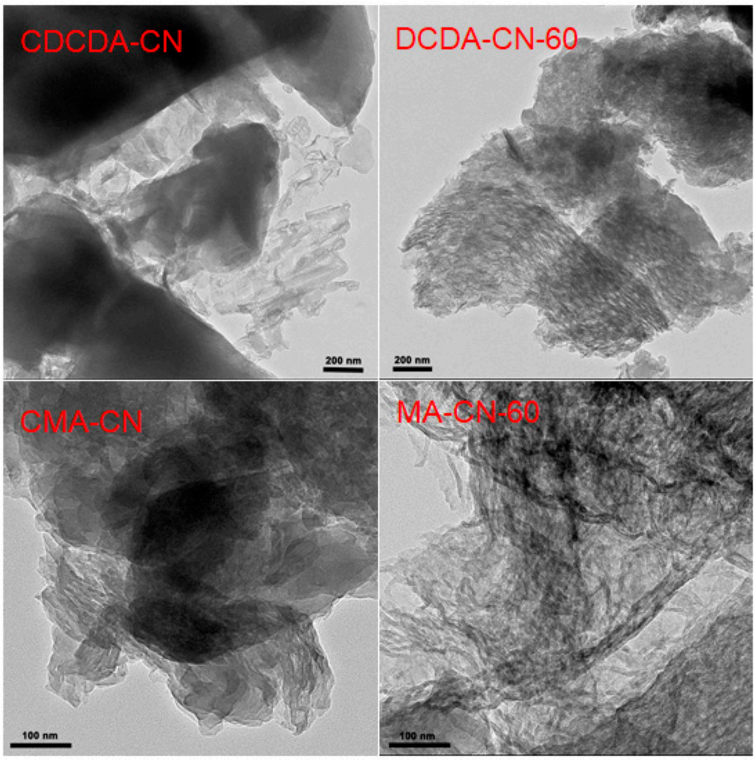

Figure S8. TEM images of CDCDA-CN, DCDA-CN-60, CMA-CN, and MA-CN-60.

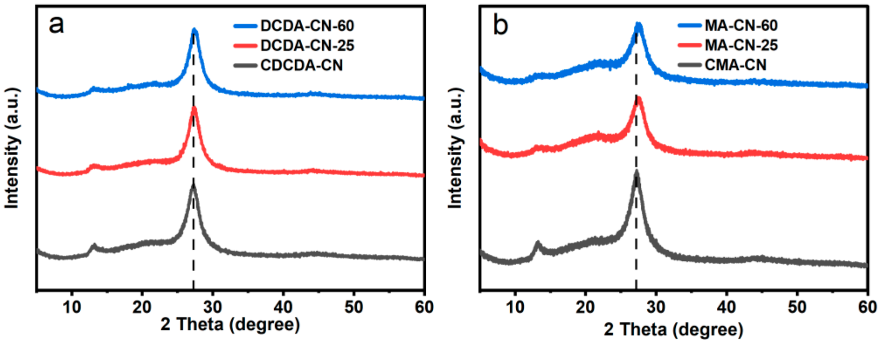

Figure S9. XRD patterns of CDCDA-CN and DCDA-CN-x (a), CMA-CN and MA-CN-x (b).

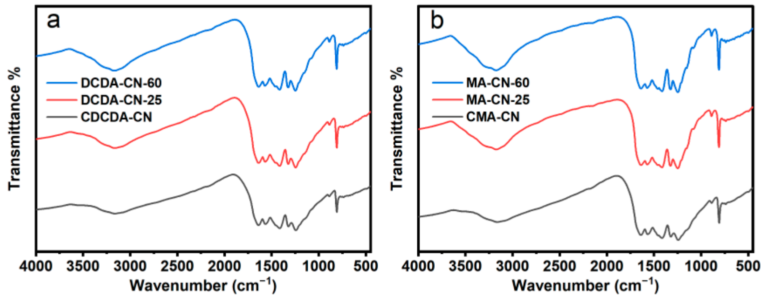

Figure S10. FTIR spectra of CDCDA-CN and DCDA-CN-x (a), CMA-CN and MA-CN-x (b).

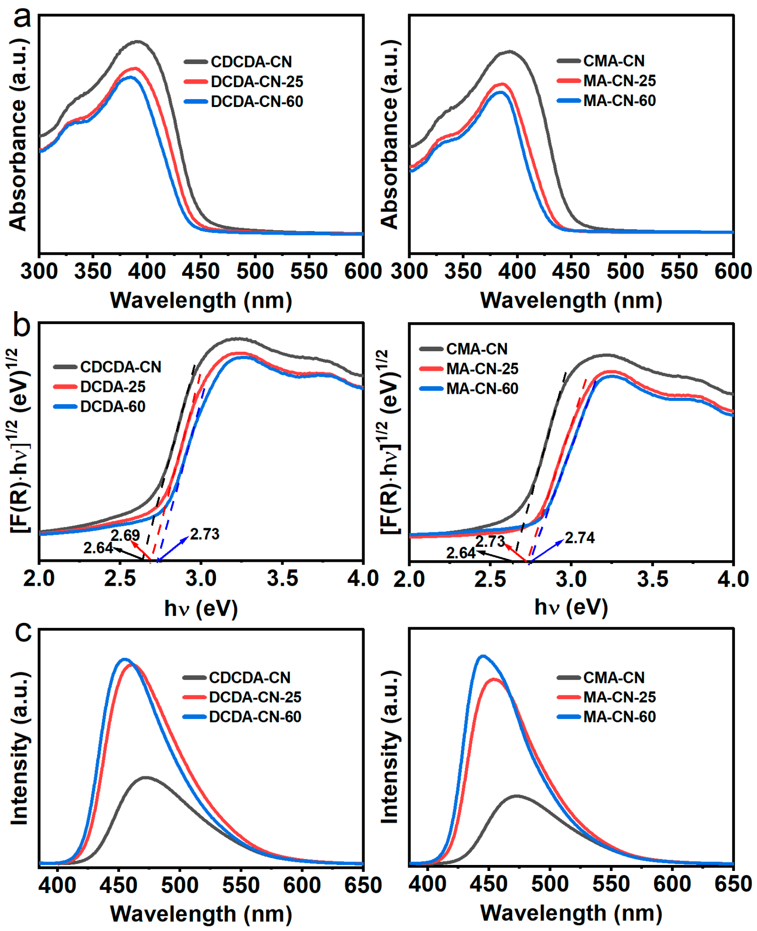

Figure S11. UV-Vis diffuse reflectance spectra (a), the corresponding Kubelka-Munk transformed spectra (b) and PL spectra of CDCDA-CN, DCDA-CN-x, CMA-CN and MA-CN-x (c).

Table S1. Surface atomic ratio of all photocatalysts determined by XPS spectra

Photocatalyst	C (at. %)	N (at. %)	O (at. %)	C/N atomic ratio
CGS-CN	40.89	55.57	3.54	0.736
GS-CN-25	40.67	56.59	2.79	0.719
GS-CN-60	40.62	56.87	2.46	0.714
GS-CN-25-4h	40.67	56.73	2.6	0.717

Table S2. The atom percentage of C, N, O and H atoms in the CGS-CN and GS-CN-60 photocatalysts determined by EA

Photocatalyst	C (wt. %)	N (wt. %)	O (wt. %)	H (wt. %)	C/N atomic ratio
CGS-CN	33.32	57.38	7.03	2.27	0.677
GS-CN-25	32.71	57.32	7.64	2.33	0.666
GS-CN-60	32.15	56.50	8.92	2.43	0.664
GS-CN-25-4h	31.76	55.50	10.16	2.58	0.668

Table S3. Lifetime and Relative Intensities of the fitting parameters of PL decay curves for CGS-CN and GS-CN-x photocatalysts

Photocatalyst	τ_1 (ns)	Intensity1 (%)	τ_2 (ns)	Intensity2 (%)	τ_{ave} (ns)
CGS-CN	1.75	33.62	9.18	66.38	6.68
GS-CN-25	1.83	35.17	8.74	64.83	6.31
GS-CN-60	1.73	35.37	7.45	64.63	5.42
GS-CN-25-4h	1.76	39	7.84	61	5.47

Table S4. BET specific surface areas (S_{BET}) of the photocatalysts

Photocatalyst	S_{BET}/$m^2 \cdot g^{-1}$
CDCDA-CN	9.6
DCDA-CN-25	12.5
DCDA-CN-60	17.1
CMA-CN	0.4
MA-CN-25	20.7
MA-CN-60	36.8
